# Mechanism underlying the acceleration of pitting corrosion of B30 copper–nickel alloy by *Pseudomonas aeruginosa*

**DOI:** 10.3389/fmicb.2023.1149110

**Published:** 2023-04-26

**Authors:** Huan Li, Mingxian Sun, Min Du, Zhenxu Zheng, Li Ma

**Affiliations:** ^1^The Key Laboratory of Marine Chemistry Theory and Technology, Ministry of Education, College of Chemistry and Chemical Engineering, Ocean University of China, Qingdao, China; ^2^State Key Laboratory for Marine Corrosion and Protection, Luoyang Ship Material Research Institute (LSMRI), Qingdao, China

**Keywords:** B30 copper–nickel alloy, *Pseudomonas aeruginosa*, accelerated pitting, passivation film, microbial corrosion

## Abstract

Despite its excellent corrosion resistance, B30 copper–nickel alloy is prone to pitting, particularly when exposed to microorganisms. The mechanism underlying the acceleration of pitting in this alloy is not fully understood. In this study, the acceleration of pitting corrosion in B30 copper–nickel alloy caused by a marine microorganism named *Pseudomonas aeruginosa* (*P. aeruginosa*) was investigated using surface analysis and electrochemical techniques. *P. aeruginosa* significantly accelerated the pitting in B30 copper–nickel alloy, with a maximum pitting depth of 1.9 times that of the abiotic control and a significant increase in pitting density. This can be attributed to extracellular electron transfer and copper–ammonia complex production by *P. aeruginosa*, accelerating the breakdown of the passivation film.

## 1. Introduction

B30 copper–nickel alloy is widely used for manufacturing marine condensers owing to its excellent resistance to seawater corrosion, scouring corrosion, and marine biological fouling (Duranceau et al., 1999; Cincera et al., 2012). However, B30 copper–nickel alloys can still experience significant pitting corrosion during service (Ilhan-Sungur and Çotuk, 2010). Both the poor surface film conditions and harsh environment are likely to cause severe pitting corrosion. Kong found that passivation film defects increase with temperature, with a higher probability of passivation breakdown at higher temperatures and more susceptible to pitting (Kong et al., 2017). In the complex environment of seawater condensing systems, various factors such as pH, dissolved oxygen, chlorine, and flushing rate may contribute to the pitting. However, studies have shown that these conditions do not significantly accelerate the pitting of B30 copper–nickel alloy (Ismail et al., 2006; Metikoš-Huković et al., 2011; Zhang et al., 2014; Sun et al., 2015). Microorganisms, exhibiting a high level of diversity and adaptability, are prevalent in natural and industrial environments. They are capable of causing corrosion in almost all types of metals and alloys (Galarce et al., 2019; Gu et al., 2019). The operating environment that condensing systems provide is conducive to the growth and reproduction of microorganisms (Aruliah and Ting, 2014). Microbial metabolism produces inorganic acids, organic acids, sulfides, ammonia, and other substances that enhance the corrosiveness of the environment (Agarwal, 2002a; Yuan and Pehkonen, 2007). Microbial reproduction and metabolism also change the oxygen concentration, salinity, acidity, and other environmental conditions surrounding the metal; thus, contributing to further corrosion in the metal (Enning and Garrelfs, 2014).

*Pseudomonas aeruginosa* (*P. aeruginosa*) is widely distributed in various environments because of its unique physiological and metabolic properties and environmental adaptability. It is frequently found in steel failures in marine environments. Its impact on the corrosion of metallic materials has been gradually attracting attention. Hamzah et al. (2014) studied the corrosion behavior of carbon steel in seawater medium with *P. aeruginosa*. The weight loss results show that the corrosion rate of steel substrates in bacteria-contained medium was around 1.6 times higher than corrosion rate of steel substrates in sterile medium indicating the aggressive role of bacteria on corrosion acceleration. Lou et al. (2020) found that compared to the sterile control, the formation of *P. aeruginosa* biofilms induced more severe localized corrosion on the FeCoCrNiMo_0_._1_ high-entropy alloys (HEA) surface. Pu et al. (2020) reported that *P. aeruginosa* caused copper corrosion through extracellular electron transfer. *P. aeruginosa* is a gram-negative facultative aerobic bacterium, a typical nitrate-reducing bacterium. It can reduce nitrate to NH_4_^+^ or N_2_ by the action of enzymes to obtain electrons (Li et al., 2017). Initially, *P. aeruginosa* was considered the pioneer colonizing bacterium in the biofilm formation process by consuming oxygen and providing an anaerobic environment for other anaerobic corrosive bacteria, such as sulfate-reducing bacteria (Batmanghelich et al., 2017). Subsequent research revealed that these strains are aerobic slime-forming bacteria that usually grow in patchy distributions on metal surfaces. They exclude oxygen through respiration, creating an oxygen-concentration cell or ion-concentration cell (Gubner and Beech, 2000). The metabolites secreted by *P. aeruginosa* can promote the rupture of passivation films on metal surfaces, leading to the accelerated dissolution of metal substrates (Xu et al., 2018; Zhou et al., 2018). Li et al. (2017) investigated the effect of *P. aeruginosa* on the microbiologically influenced corrosion (MIC) of stainless steel. They found that *P. aeruginosa* caused the oxidation and dissolution of alloying elements and catalyzes the generation of soluble CrO_3_ complexes, instead of forming passive chromium oxides such as Cr_2_O_3_, therefore accelerating the onset of pitting. *P. aeruginosa* is capable of secreting a series of redox-active phenazines, which are secondary metabolites composed of nitrogen-containing heterocyclic pigment compounds (Ren et al., 2018). These phenazines can act as electron carriers for the extracellular electron transfer of *P. aeruginosa*, balancing the intracellular redox state. Furthermore, they have an impact on the biofilm-forming ability of *P. aeruginosa* (Huang et al., 2018). The effect of *P. aeruginosa* on the corrosion in metal materials such as carbon steel, stainless steel, and titanium has been studied extensively (Mahat et al., 2012; Lou et al., 2016; Saleem Khan et al., 2019; Huang et al., 2020), but few studies focused on the *P. aeruginosa* MIC of B30 copper–nickel alloy. However, a large population of both anaerobic and aerobic bacteria, especially *Pseudomonad* genus, were found in the condenser (Beech and Sunner, 2006).

Therefore, given the severe pitting problems in copper-nickel alloys under actual working conditions, it is urgent to study the key role of *P. aeruginosa* in the pitting corrosion of B30 copper–nickel alloy. In this study, surface analysis and electrochemical measurement techniques were used to investigate the formation and development pattern of pitting corrosion in B30 copper–nickel alloy in relation to the growth and metabolites of *P. aeruginosa*. The aim of this study was to reveal the mechanism behind the rapid pitting corrosion in B30 copper–nickel alloy caused by *P. aeruginosa*.

## 2. Materials and methods

### 2.1. Preparation of specimens

B30 copper–nickel alloy was provided by Xinyang Shengxin Technology Co., Ltd. of Shandong Province. The composition of B30 copper–nickel was as follows: Cu 65.8–68.6, Ni 30.0–32.0, Fe 0.6–1.0, Mn 0.5–1.0, C 0.04, Pb ≤ 0.01, S ≤ 0.01, Zn 0.2, and *P* ≤ 0.01. The electrochemical test sample was B30 copper–nickel alloy welded to a copper wire and then installed in an epoxy resin with an exposure area of 1 cm^2^. Sheet specimens with dimensions of 10 mm × 10 mm × 3 mm were used for confocal laser scanning microscopy (CLSM), scanning electron microscopy (SEM), and X-ray photoelectron spectroscopy (XPS) analysis. The samples were sequentially polished with 400, 800, 1,000, and 2,000 mesh sandpaper to ensure a consistent surface. The samples were then cleaned with deionized water, immersed in anhydrous ethanol for 15 min, and placed under ultraviolet light for 20 min before experiments were carried out.

### 2.2. Microbial cultivation

*P. aeruginosa* (PAO1 wide-type strain) was procured from Rizhao Biotechnology (Qingdao). *P. aeruginosa* was cultivated in an LB-NO_3_ seawater medium consisting of 10 g L^–1^ KNO_3_, 10 g L^–1^ tryptone, 5 g L^–1^ yeast extract, and 5 g L^–1^ NaCl in filtered seawater from Qingdao wheat island waters. The pH of the solution was adjusted to 7.2 ± 0.1 using NaOH, filled with nitrogen gas for 40 min to remove oxygen, then the media were autoclaved at 121^°^C for 20 min. Yeast extract was purchased from Thermo Fisher Scientific, while other reagents were purchased from Sinopharm Chemical Reagent Co. The concentration of planktonic *P. aeruginosa* in the solution during a growth period of 21 days was determined using the plate count method. An agar medium was used, and the samples were diluted with sterile distilled water in three gradients. 0.1 mL of the solution sample was absorbed and added to the sterile Petri dish for uniform coating. Each gradient consisted of three parallel samples. The number of bacterial colonies was calculated after incubation at 37^°^C for 48 h. Plates with an average number of colonies between 30 and 300 were selected for counting. The pH of the culture medium was measured in triplicate with a pH meter (E-201-C, LEICI, Shanghai, China), and the NH_4_^+^ concentration in solution was determined by using an ammonium ion selective electrode (NH_4_-US, BANTE, Shanghai, China).

### 2.3. Surface characterization

After being immersed in *P. aeruginosa* inoculation medium at 25^°^C for 7, 14, and 21 days, the samples’ pit morphology and maximum pit depth were observed via CLSM (vk-x250k type, Keyence, Osaka, Japan). Samples were treated by ultrasonic cleaning in absolute ethanol for 15 min to remove biofilm and then subsequently treated with 10% dilute sulfuric acid for 1 min to remove corrosion products.

SEM (Ultra Plus, Zeiss, Germany) was used to examine the biofilm and pit morphology on the sample surface. First, the sample was submerged in a 5% (w/w) glutaraldehyde solution for 2 h to kill and immobilize the biofilm. Then the biofilm was dehydrated sequentially in 30, 50, 70, 90, and 100% (v/v) isopropanol concentration for 5 min each. After, the specimens were blow-dried with N_2_. To enhance the electrical conductivity, a gold film was sprayed on the surface of the specimen before the SEM measurements (Xu et al., 2013).

The corrosion products composition were analyzed using XPS (ESCALAB 250Xi, Thermo VG, USA). After being gently washed in phosphate-buffered saline solution, the specimens were blow-dried with N_2_ and sealed. The photoelectrons were excited with an Al Kα (1486.6 eV) X-ray source, and the analyzer passed at an energy of 30.0 eV. Binding energies were calibrated against surface carbon contamination at 284.8 eV. Composition-depth profiles were obtained using 2.0 keV argon ions at a target current of 2.0 μA cm^–2^ and a pressure of 7.7 × 10^–9^ MPa. The etching rate was approximately 0.25 nm s^–1^ (vs. Ta_2_O_5_), and the etching time was 2,000 s (Ma et al., 2015).

### 2.4. Electrochemical measurements

The electrochemical test used a three-electrode system, with B30 copper–nickel alloy as the working electrode, the noble metal oxide electrode (MMO) as the counter electrode, and a saturated calomel electrode (SCE) as the reference electrode. The open-circuit potential (OCP), electrochemical impedance spectroscopy (EIS), and kinetic potential anodic polarization curves were determined using a Gamry constant potential instrument (Reference 600, Gamry Instruments, Warminster, PA, USA). EIS was performed by applying a sinusoidal voltage of 10 mV in a frequency range of 10^–2^ to 10^5^ Hz at a stable OCP, and the resulting data were fitted and analyzed using the ZView2 software. Each working electrode was measured once in a scan range of 0 to 800 mV vs. OCP and 0.334 mV s^–1^ to obtain the anodic polarization curve.

All of the above tests were performed at 25^°^C and repeated three times to verify the reproducibility of the data.

## 3. Results

### 3.1. Cell count, pH value, and NH_4_^+^ concentration

**FIGURE 1:**
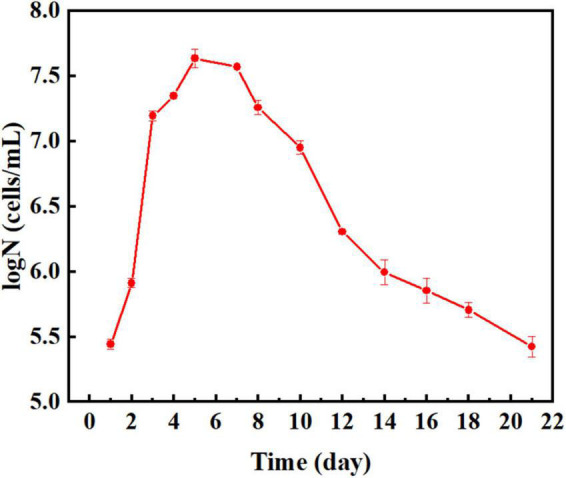
Planktonic cell counts of *P. aeruginosa* at 21 days of culture.

Figure 1 shows the growth curve of *P. aeruginosa* obtained by continuous incubation for 21 days. The population of *P. aeruginosa* increased continuously during the first 5 days, culminating a maximum of 4.47 × 10^7^ cells mL^–1^ at the end of this period. The copper–nickel alloy gradually released copper ions, which can be toxic to bacteria at high concentrations. As nutrients were depleted in the medium, the population of *P. aeruginosa* entered the phase of decline and decreased continuously after 5 days. However, some bacteria were able to survive until the end of the 21 days incubation period.

**FIGURE 2:**
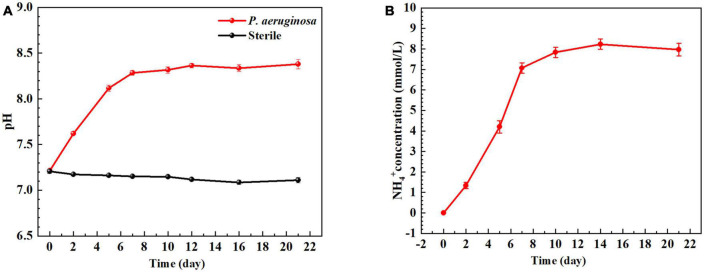
**(A)** pH value and **(B)** NH_4_^+^ concentration of B30 copper–nickel alloy during immersion.

Figure 2A shows the change in the pH value of the B30 copper-nickel sample immersed in the sterile system and *P. aeruginosa* for 21 days. In the sterile system, the pH value is stable at 7.2. In *P. aeruginosa*, the pH value first rapidly increases and then stabilizes, possibly because of the reduction of NO_3_^–^ to NH_4_^+^ by *P. aeruginosa*. Figure 2B shows the change in NH_4_^+^ concentration in *P. aeruginosa*. For the initial 7 days, the concentration of NH_4_^+^ rapidly increases because the life activity of *P. aeruginosa* is vigorous and the number of bacteria increases.

### 3.2. Pitting phenomenon

**FIGURE 3:**
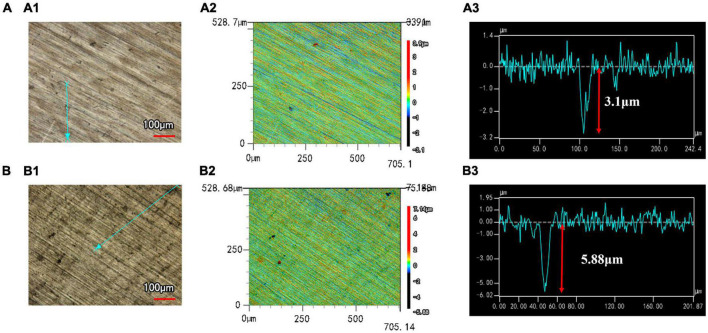
Corrosion morphology and the maximum pitting depth of B30 copper–nickel alloy after immersion in different media for 21 days: **(A)** abiotic medium; **(B)** *P. aeruginosa*.

Figure 3 presents the 3D morphology and the maximum pit depth of B30 copper–nickel alloy as measured by CLSM. The B30 copper–nickel alloy sample exhibited a maximum pit depth of 3.1 μm after being immersed in an abiotic medium for 21 days. In contrast, the sample immersed in *P. aeruginosa* had a maximum pit depth of 5.88 μm, which was 1.90 times larger than that of the abiotic control. Moreover, a larger number of pits were observed on its surface.

To compare the pit size in different media and assess the effect of *P. aeruginosa* on pitting development, a detailed statistical analysis of pit diameter and pit depth was performed. 20 pits on each surface were selected, and the cumulative probability (*P*_*cum*_) of the pits was calculated, along with the average pit size and standard deviation. The cumulative probability (*P*_*cum*_) was calculated using the average rank method, which is defined as *P_*cum*_* = *i*/(1 + *N*), where *i* represents the order of the total number of pits and *N* is the total number of pits actually measured (Biesinger et al., 2010; Cui et al., 2014).

**FIGURE 4:**
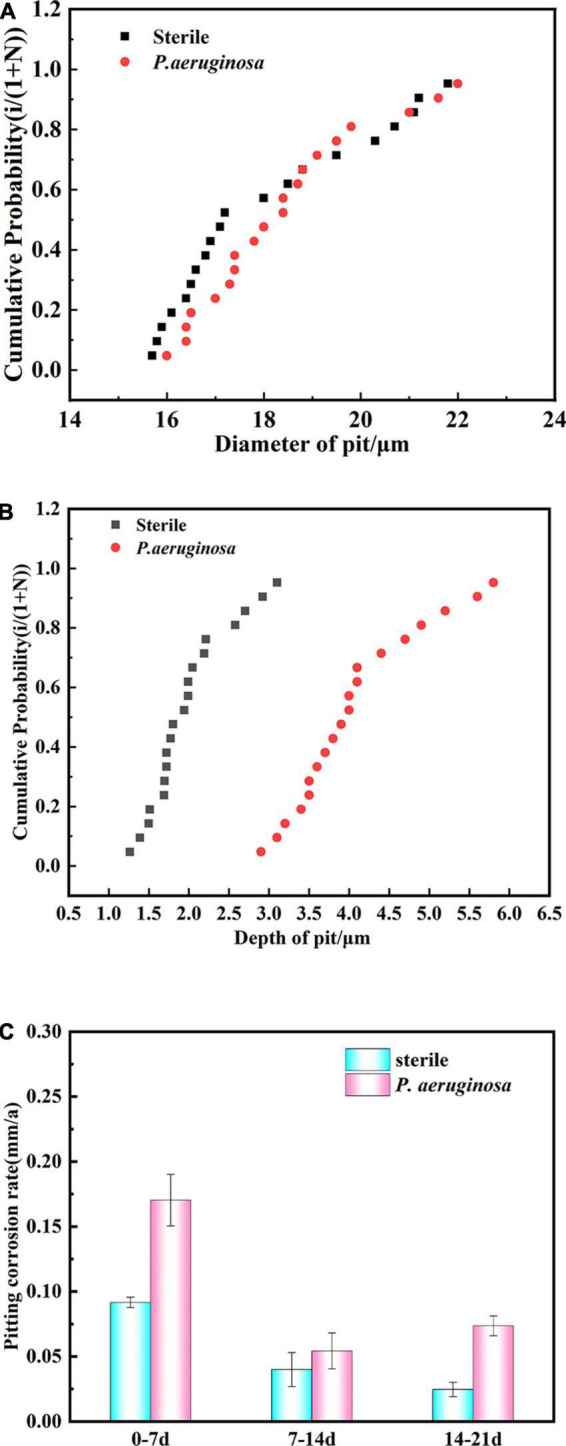
Statistical distribution of **(A)** pit diameter and **(B)** pit depth of B30 copper–nickel alloy immersed in abiotic medium and *P. aeruginosa* for 21 days. **(C)** Pitting rate at different times of immersion.

Figure 4A shows the statistical distribution of pit diameters observed on copper–nickel alloy specimens immersed in different media for 21 days. The pit diameters of the specimens were concentrated between 16.0 and 22.0 μm for the abiotic control and *P. aeruginosa*, which showed no significant variation in either. Figure 4B shows the statistical distribution of the depths of B30 copper–nickel alloy pits in abiotic medium and *P. aeruginosa* during the immersion test. The pit depths on the specimen surfaces in the abiotic medium were primarily concentrated between 1.2 and 3.0 μm, while those in *P. aeruginosa* were concentrated between 3.0 and 5.8 μm, significantly deeper than those in the abiotic control. The presence of *P. aeruginosa* increased the pit depth of the copper–nickel alloy compared to the abiotic control specimens.

B30 copper–nickel alloy was immersed in the abiotic control and *P. aeruginosa* for various periods of time, and the maximum pit depth was statistically analyzed using CLSM. The calculated pitting rate is shown in Figure 4C. The highest pitting rate of the specimens was primarily concentrated in the early stage of immersion (0–7 days). The pitting rate of the abiotic control was 0.092 mm a^–1^, while that of the specimens in *P. aeruginosa* was 0.170 mm a^–1^, 1.85 times higher than that in the abiotic control. At the end stage of immersion (14–21 days), the pitting rate of the specimens in *P. aeruginosa* demonstrated an increasing trend of 0.074 mm a^–1^, which was 2.96 times higher than that of the abiotic control.

### 3.3. Surface topography and corrosion product composition

**FIGURE 5:**
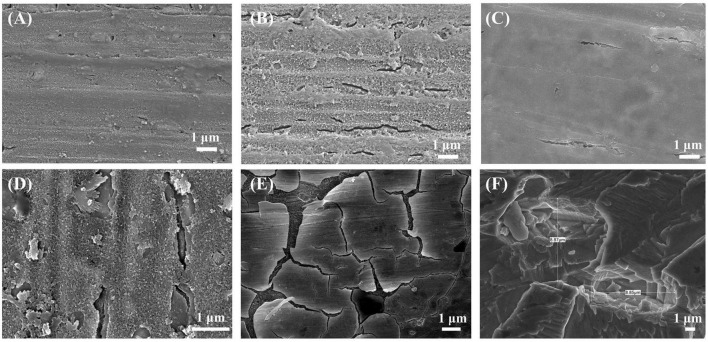
Corrosion morphology of B30 copper–nickel alloy after immersion in the abiotic medium for **(A)** 7 days and **(B)** 14 days and in *P. aeruginosa* for **(C)** 7 days, **(D)** 14 days, and **(E)** 21 days; **(F)** substrate after immersion for 21 days to remove corrosion products.

Figure 5 shows the SEM images of B30 copper–nickel alloy specimens immersed in abiotic and biotic media for different periods of time. After immersion in the abiotic medium for 7 days, a dense film layer on the surface of the specimen was observed (Figure 5A). The specimens that were immersed for 14 days showed evidence of a raptured surface film with visible shallow cracks (Figure 5B). The specimens that were immersed in *P. aeruginosa* for 7 days displayed surface film rupture in certain areas (Figure 5C). After 14 days of immersion, the surface film ruptured significantly, resulting in deeper cracks. The film layer appeared as a double-layer structure, consisting of a loose and porous outer layer and a dense inner layer (Figure 5D). After 21 days of immersion, the surface film layer of the copper–nickel alloy was ruptured over a large area, forming an irregular structure with a tendency to detach (Figure 5E). The corrosion products were removed from the specimens, and the substrate was observed (Figure 5F). Numerous pits were observed randomly distributed on the surface of the specimens, indicating that severe pitting in the copper–nickel alloy occurred under the conditions of immersion in *P. aeruginosa*.

**FIGURE 6:**
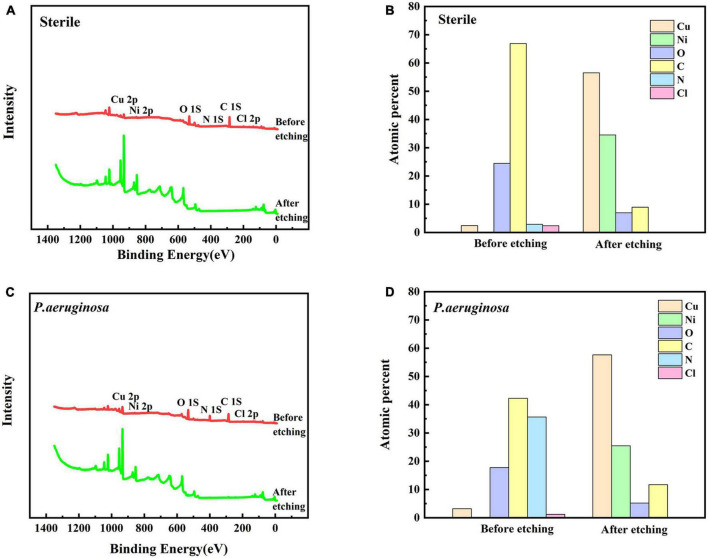
X-ray photoelectron spectroscopy (XPS) profiles **(A)** and **(C)** of B30 copper–nickel alloy immersed in different media for 21 days before and after sputtering, and depth distribution of elements on the film surface **(B)** and **(D)**.

Figures 6A, C show the XPS spectra of B30 copper–nickel alloy after 21 days of immersion in the abiotic medium and *P. aeruginosa* for surface films before and after etching, respectively. Based on the positions of the peaks, the main elements on the surface of the specimen included Cu, Ni, O, C, N, and Cl. The depth distribution of the major elements in the film layer before and after etching is shown in Figures 6B, D. Before etching, the film surface contained a large amount of C and O, a small amount of Cu, N, and Cl, and no Ni. After etching, the content of Cu and Ni increased significantly, while that of other elements decreased. Compared to the non-biological control, the film layer surface of the specimens in *P. aeruginosa* contained more N elements after etching. This is because of the attachment of *P. aeruginosa* metabolites to the surface of B30 copper–nickel alloy. To gain insight into the valence states of the internal constituent elements at different depths of the film layer, the fine spectra of each element were fitted to the split peaks. No Ni signal was detected on the surface of the specimen before etching; therefore, the Ni 2p spectrum was fitted only after etching.

**FIGURE 7:**
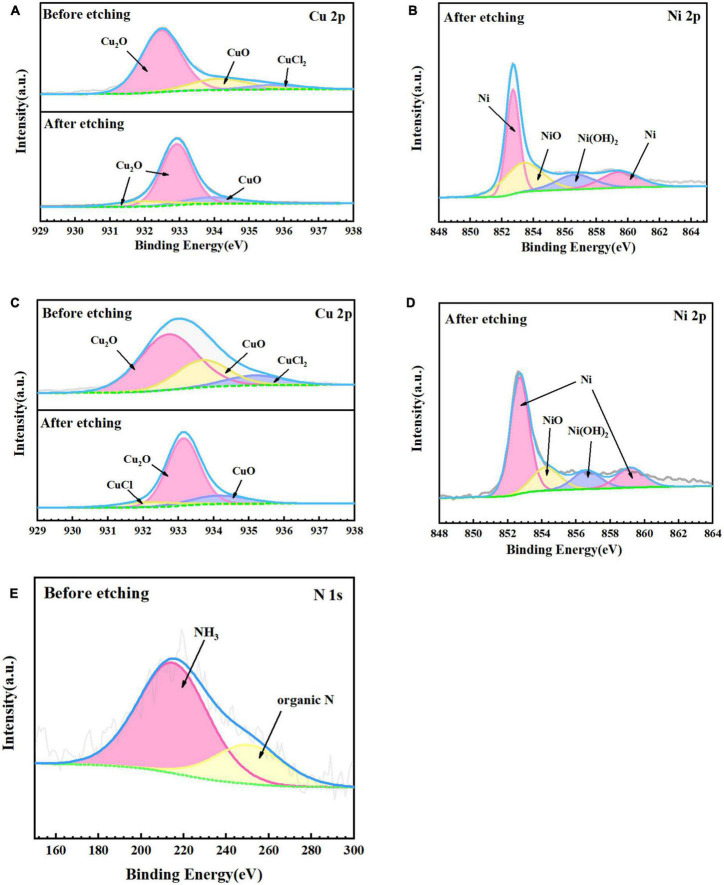
X-ray photoelectron spectroscopy (XPS) fine spectra of B30 copper–nickel alloy before and after etching after immersed in different media for 21 days: abiotic medium **(A)** Cu 2p and **(B)** Ni 2p; *P. aeruginosa* **(C)** Cu 2p, **(D)** Ni 2p, and **(E)** N 1s.

Figure 7 shows the fine spectra of corrosion product layers Cu 2p, Ni 2p, and N 1s of B30 copper–nickel alloy immersed in different media for 21 days. The specific fitting parameters are shown in Table 1. In the high-resolution XPS spectrum of Cu 2p in the sterile medium (Figure 7A), the binding energies before etching are 932.5, 934.2, and 935.6 eV, corresponding to Cu_2_O, CuO, and CuCl_2_, respectively, and only Cu_2_O and CuO are observed after etching. Ni signal is not detected before etching due to the enrichment of Ni and its compounds in the inner surface layer, which was also proved by Ma et al. (2015). The peaks at Ni 2p binding energies of 852.7 and 859.5 eV after etching are Ni, and the peaks at 853.4 and 856.6 eV are NiO and Ni(OH)_2_, respectively (Figure 7B). Cu_2_O, CuO, and CuCl_2_ are also present in the sample immersed in *P. aeruginosa* (Figure 7C) before etching, but a small amount of CuCl, a relatively loose substance, is found in the Cu 2p spectrum after etching. This indicates that B30 copper–nickel alloy forms a more porous film in the presence of *P. aeruginosa*. Two peaks appear on the N 1s spectrum (Figure 7E), namely, NH_3_ and organic N, and the metabolite of *P. aeruginosa* attaches to the sample surface.

**TABLE 1:** Attribution of surface element peaks of B30 copper–nickel alloy after 21 days of immersion in *P. aeruginosa*.

		Element	Assignment	B.E. (EV)	Content (%)
Sterile	Before etching	Cu	Cu_2_O	932.5	73.54
			CuO	934.2	19.13
			CuCl_2_	935.6	7.33
	After etching	Cu	Cu_2_O	932.9/932.0	84.58
			CuO	934.0	15.52
		Ni	Ni	852.7/859.5	54.10
			NiO	853.4	30.48
			Ni(OH)_2_	856.6	15.42
*P. aeruginosa*	Before etching	Cu	Cu_2_O	932.7	61.86
			CuO	933.6	27.20
			CuCl_2_	935.2	10.94
		N	NH_3_	399.6	74.01
			Organic N	397.7	25.99
	After etching	Cu	Cu_2_O	933.1	73.94
			CuO	934.3	16.23
			CuCl	932.1	9.83
		Ni	Ni	852.7/859.2	70.45
			NiO	854.3	16.59
			Ni(OH)_2_	856.6	12.96

**FIGURE 8:**
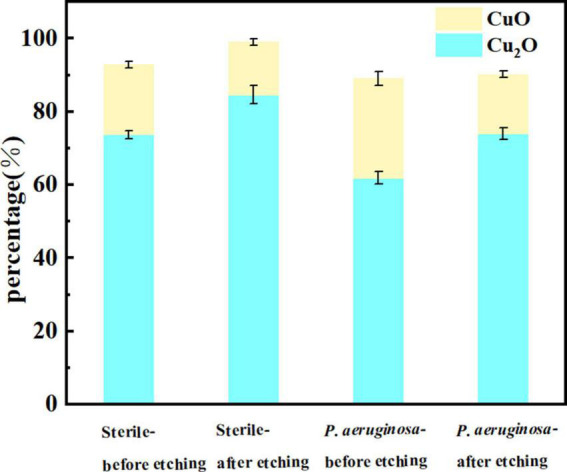
Cu oxide content on the surface of B30 copper–nickel alloy after 21 days of immersion in different media.

Figure 8 presents the oxide compositions of different Cu valences extracted from the fitted results. The excellent corrosion resistance of B30 copper–nickel alloy is attributed to the formation of dense Cu_2_O, while loose and porous CuO is not protective. The specimens immersed in non-biological media exhibited 19.13% CuO before etching and 14.12% CuO after etching. In contrast, specimens immersed in *P. aeruginosa* exhibited a CuO content of 27.20% before etching and 16.23% after etching. This indicates that *P. aeruginosa* rendered B30 copper–nickel alloy more prone to the generation of the unprotective corrosion product CuO.

### 3.4. Corrosion electrochemical behavior

**FIGURE 9:**
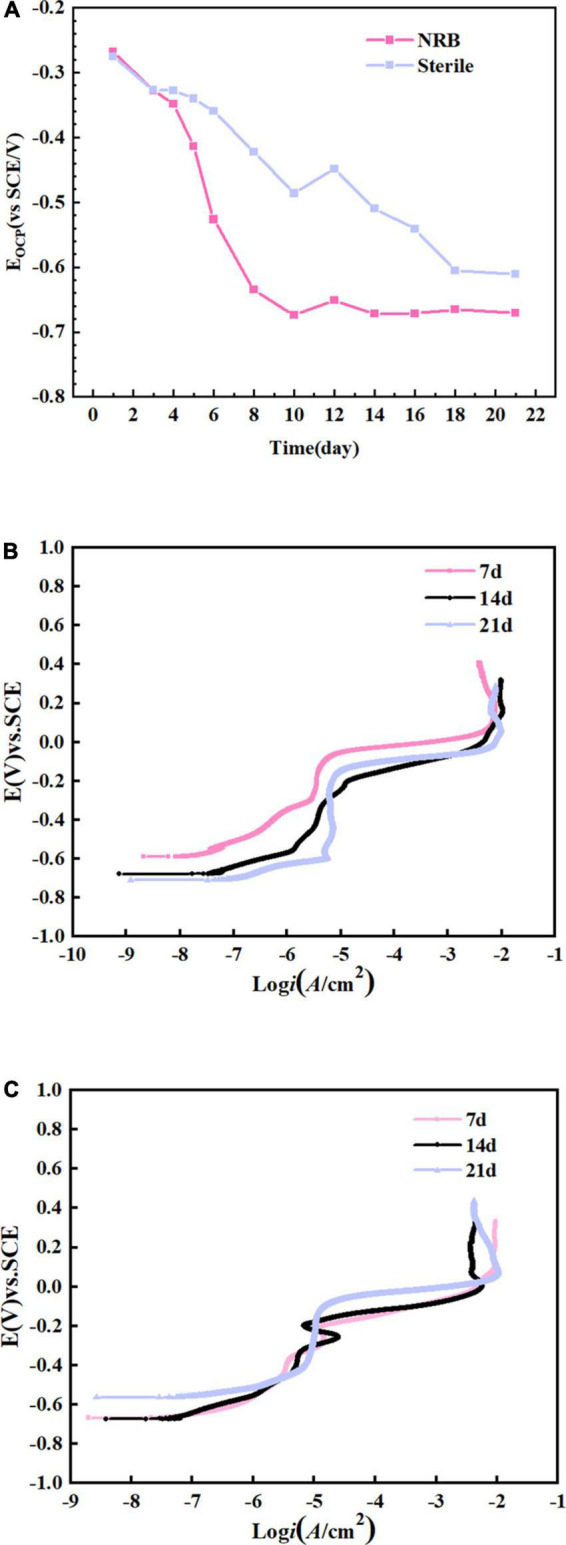
B30 copper–nickel alloy specimens in abiotic medium and *P. aeruginosa*: **(A)** open-circuit potentia (OCP) and **(B,C)** anodic polarization curves.

Figure 9A shows the change in OCP of the specimens immersed in *P. aeruginosa* and the abiotic medium for 21 days. The OCP for specimens immersed under abiotic and biotic conditions became increasingly negative with increasing immersion time. A comparison of the two revealed that the OCP of the B30 copper–nickel alloy in *P. aeruginosa* was much lower than that of the abiotic control. Figures 9B, C show the anodic polarization curves of the electrochemical specimens of B30 copper–nickel alloy immersed in different media for 7, 14, and 21 days. The anodic polarization curves in the abiotic medium, indicate that the passivation zone increased with increasing immersion time. The passivation zone on the anodic polarization curve of *P. aeruginosa* was significantly smaller compared to the abiotic control. This could be due to depletion of Cu_2_O in the environment with *P aeruginosa*. After 14 days of immersion, when B30 copper-nickel alloy sample in NRB gradually approached the pitting potential with the increase of potential, there is a small current fluctuation in the passivation zone, which is due to the formation of metastable pitting corrosion. When the solution potential reached the breakdown potential, the current density rose sharply, indicating the formation of steady-state pitting pits on the sample surface.

Table 2 shows the statistical analysis of the pitting potential (*E*_*pit*_), OCP (*E*_*corr*_), and pitting nucleation resistance (*E*_*pit*_–*E*_*corr*_) on the anodic polarization curve. As shown in the table, the specimens in *P. aeruginosa* exhibited an overall more negative pitting potential and less resistance to pitting nucleation compared to the abiotic control. This indicates a higher susceptibility to pitting and a greater tendency to sprout pitting.

**TABLE 2:** Open-circuit potential (OCP) and pitting potential obtained after the anodic polarization curve test.

	Time (d)	E_corr_ (V vs. SCE)	E_p_ (V vs. SCE)	E_*p*_-E_corr_ (V vs. SCE)
Sterile	7	−0.589	−0.079	0.510
	14	−0.678	−0.199	0.479
	21	−0.709	−0.174	0.535
*P. aeruginosa*	7	−0.667	−0.206	0.461
	14	−0.673	−0.195	0.478
	21	−0.563	−0.138	0.425

**FIGURE 10:**
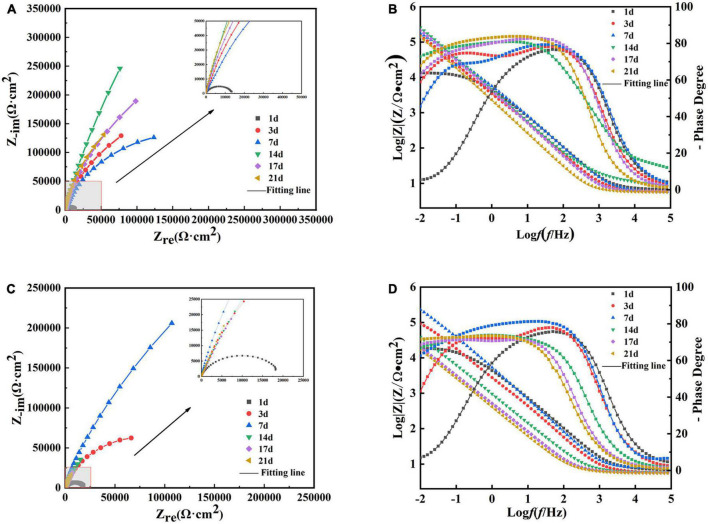
Electrochemical impedance spectra of B30 copper–nickel alloy in different media: **(A)** Nyquist and **(B)** Bode plots of specimens in abiotic medium and **(C)** Nyquist and **(D)** Bode plots of specimens in *P. aeruginosa*.

**FIGURE 11:**
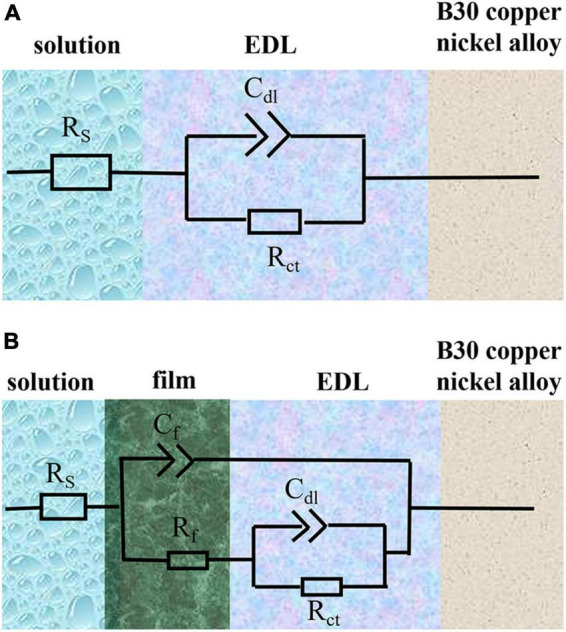
Equivalent electrical circuits used to fit the electrochemical impedance spectra data. *R*_s_, solution resistance; *C*_dl_, capacitance of electric double layer (EDL); *R*_ct_, charge transfer resistance; *C*_f_, capacitance of biofilm; *R*_f_, surface film (including biofilm/corrosion product) resistance. **(A)** Shows B30 copper-nickel alloy immersed in the abiotic medium and *P. aeruginosa* at 1 day, whereas **(B)** depicts other days of immersion.

Figures 10A, B show the Nyquist and Bode plots, respectively, obtained by immersing the B30 copper–nickel alloy specimens in the abiotic medium. The radius of the capacitive arc increases with immersed time, and after 14 days, the capacitive arc starts to decrease. Figures 10C, D show the Nyquist and Bode plots of B30 copper–nickel alloy immersed in *P. aeruginosa*, respectively. An overall increase trend followed by a decrease in the capacitive arc was observed. Although consistent with the trend of the abiotic control, the capacitive arc of the specimens in *P. aeruginosa* started to show a decreasing trend at 7 days. Furthermore, the phase angle peak was significantly narrower, indicating that the film layer formed under the action of *P. aeruginosa* was not sufficiently dense, and the film layer was being destroyed at an accelerated rate. According to the surface film structure and EIS characteristics of B30 copper–nickel alloy in different systems when selecting the equivalent circuit diagrams, Figure 11A shows B30 copper–nickel alloy immersed in the abiotic medium and *P. aeruginosa* at 1 day, whereas Figure 11B depicts other days of immersion.

As shown by the fitted data in Table 3, in the first 5 days of the vigorous growth of *P. aeruginosa*, the *R*_*ct*_ value of the sample immersed in *P. aeruginosa* is lower than that of the non-biological control. This indicates that *P. aeruginosa* exhibits a strong ability to transfer charge on the surface of B30 copper–nickel alloy, allowing it to acquire electrons from the metal surface and gain energy.

**TABLE 3:** Electrochemical impedance spectra fitting results for B30 copper–nickel alloy in different media.

	*t* (d)	*R*_s_ (Ω⋅cm^2^)	*R*_ct_ (KΩ⋅cm^2^)	*C*_dl_ (F⋅cm^–2^)	*n*_dl_	*R*_f_ (KΩ⋅cm^2^)	*C*_f_ (F⋅cm^–2^)	*n*_f_
Sterile	1	6.55	12.83	3.77 × 10^–5^	0.86	-	–	–
	3	6.01	446.59	2.06 × 10^–5^	0.80	6.00	4.33 × 10^–5^	0.90
	5	5.81	909.20	2.30 × 10^–5^	0.91	17.28	1.16 × 10^–5^	0.76
	7	5.90	317.17	1.76 × 10^–5^	0.78	19.84	2.94 × 10^–5^	0.90
	14	4.66	2414.70	2.24 × 10^–5^	0.89	1.41	6.7 × 10^–6^	0.79
	21	5.79	551.10	1.00 × 10^–5^	0.67	96.34	7.65 × 10^–5^	0.94
*P. aeruginosa*	1	6.81	18.11	4.29 × 10^–5^	0.84	-	–	–
	3	5.85	157.87	2.99 × 10^–5^	0.78	4.09	5.23 × 10^–5^	0.90
	5	5.92	271.06	5.27 × 10^–5^	0.89	8.57	1.69 × 10^–5^	0.89
	7	6.52	1247.00	5.91 × 10^–5^	0.92	67.79	1.53 × 10^–5^	0.67
	14	5.77	135.01	9.38 × 10^–5^	0.82	142.45	2.28 × 10^–4^	0.83
	21	5.64	370.75	5.82 × 10^–5^	0.80	48.38	5.24 × 10^–4^	0.83

## 4. Discussion

It is known that the excellent corrosion resistance of B30 copper–nickel alloy lies in the generation of a passivation film Cu_2_O on the surface. As the immersion time in seawater increased, the Cu_2_O film became thicker. The outer layer of the film was formed by the re-precipitation of dissolved copper, as shown in equation (1), while the inner layer was formed by the inward growth of the Cu_2_O film, which is denser (Ma et al., 2015).


(1) $$4{\text{Cu + O}}_{2}\to 2{\text{Cu}}_{\text{2}}\text{O}$$

Ni and its compounds are mainly enriched in the inner layer of the corrosion product film (Figure 7), which enhances the passivation of B30 copper–nickel alloy. Ni forms NiO through passivation or transformation within the Cu_2_O lattice, and then NiO undergoes hydrolysis to produce Ni(OH)_2_. There is always a certain concentration of metallic Ni in the inner layer of the film, which accumulates because Ni less prone to oxidation than Cu (Hodgkiess and Vassiliou, 2005).

**FIGURE 12:**
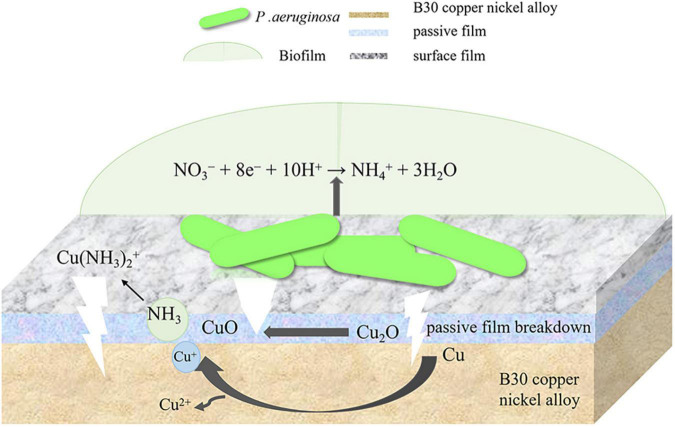
Acceleration mechanism of *P. aeruginosa* on the B30 copper–nickel alloy pitting corrosion.

Confocal laser scanning microscopy results demonstrated that the maximum pit depth of specimens after 21 days of immersion in *P. aeruginosa* (Figure 3) was 5.88 μm, 1.9 times that of the abiotic control. The pitting rate in *P. aeruginosa* was also significantly higher than that of the abiotic control as can be seen from Figure 4. The acceleration mechanism of *P. aeruginosa* on the B30 copper–nickel alloy pitting corrosion is shown in Figure 12, by the mechanism explained by the reactions below:

When nitrate acts as an electron acceptor, it can be reduced to NH_4_^+^or N_2_.


(2) $${\text{2NO}}_{3}{}^{-}+10{\text{e}}^{-}+12{\text{H}}^{+}\to {\text{N}}_{2}+{\text{6H}}_{2}{\text{O E}}_{\text{e}}=+749\text{mV}$$

(3) $${\text{NO}}_{3}{}^{-}+8{\text{e}}^{-}+10{\text{H}}^{+}\to {\text{NH}}_{4}{}^{+}+3{\text{H}}_{2}{\text{O E}}_{\text{e}}=+358\text{mV}$$

Monomeric copper can provide electrons for nitrate reduction. Its oxidation reaction and the corresponding equilibrium potential are shown below (Dou et al., 2018; Pu et al., 2020).


(4) $$\text{Cu}\to {\text{Cu}}^{+}+{\text{e}}^{-}{\text{E}}_{\text{e}}= + 520mV$$

(5) $$\text{Cu}\to {\text{Cu}}^{2+}+2{\text{e}}^{-}{\text{E}}_{\text{e}}=+340\text{mV}$$

(6) $${\text{Cu}}_{2}{\text{O + H}}_{2}\text{O}\to \text{2CuO}+2{\text{e}}^{-}+2{\text{H}}^{+}{\text{E}}_{\text{e}}=+\;669\text{mv}$$

The above equations are expressed with respect to the standard hydrogen electrode at 25^°^C, pH 7, and a solute concentration of 1 M (excluding H^+^). The electric potential for the nitrate reduction (2) and Cu oxidation reaction (4) is *E* = +229 mV. The *E* values for (2) and (5), (2) and (6), (3) and (4), (3) and (5), and (3) and (6) are +409, +80, -162,+18, and -311 mV, respectively. Except for reactions (3) and (4) and reactions (3) and (6), the *E* values for the sets of reactions are positive, and the corresponding Δ*G* is negative, indicating that these four overall reactions are thermodynamically favorable. This suggests that Cu or Cu_2_O can provide energy for nitrate respiration by *P. aeruginosa* through extracellular electron transport (Glasser et al., 2017). *P. aeruginosa* is capable of secreting a variety of redox-active phenazines, including pyocyanin (PYO) and phenazine-1-carboxamide (PCN) (Sakhtah et al., 2016; Huang et al., 2020). PYO and PCN are electron shuttles that may accelerate electron transfer between *P. aeruginosa* cells and Cu substrates. This was confirmed by the results of the EIS fit, where the R_*ct*_ values of the specimens in *P. aeruginosa* were much smaller than those of the abiotic control, which may suggest the presence of P. aeruginosa enhances the charge transfer ability of B30 copper-nickel alloy. Phenazines act as indirect electron carriers to receive electrons extracellularly that are generated from the dissolution of the Cu substrate or the transformation of Cu_2_O into CuO in MIC.

According to the SEM results, the film layer of B30 copper–nickel alloy remained dense and intact after 7 days in the abiotic medium. However, in *P. aeruginosa*, the film layer was already ruptured (Figure 5C), indicating that the presence of *P. aeruginosa* accelerates the breakdown of the passivation film. The EIS results also confirm these conclusions: the radius of the capacitive resistance arc in the Bode plot decreases at 14 days in the abiotic control and at 7 days in *P. aeruginosa*. The XPS results further indicate that *P. aeruginosa* induces the conversion of the protective Cu_2_O to CuO. The rupture of the passivation film creates a nucleation point for pitting, this allows the metal substrate under the film to interact with the surrounding environment and enter an activated state. Under these conditions, a small anode–large cathode (activation–passivation) corrosion cell is formed (Soltis, 2015), and pitting occurs. Pitting nucleation resistance was lower in the presence of *P. aeruginosa*, indicating a high susceptibility to pitting and a greater tendency to sprout pitting. The data in Table 2 provide evidence for this. The copper substrate at the bottom of the pitting pit continues to lose electrons as an anode, and reduction of nitrate by *P. aeruginosa* promotes the dissolution of copper. Cu^+^ is generated and then diffuses outward to react with the corrosive ion Cl^–^ to produce copper chloride. This causes pitting to develop vertically and the rate of pitting increases at the end stage of immersion (Figure 3C).


(7) $$\text{Cu}+{\text{2NH}}_{3}\to \text{Cu}{\left({\text{NH}}_{3}\right)}_{2}{}^{+}+{\text{e}}^{-}$$

(8) $${\text{Cu}}_{2}{\text{O + H}}_{2}{\text{O + 8NH}}_{3}\to \text{2}{\left[\text{Cu}{\left({\text{NH}}_{3}\right)}_{4}\right]}^{2+}+{\text{2OH}}^{-}+2{\text{e}}^{-}$$

B30 copper–nickel alloy is highly susceptible to corrosion in the presence of ammonia (Agarwal, 2002a), particularly in the empty pumping area of the condensation line, where ammonia tends to accumulate. It can be seen from Figure 2B that NH_4_^+^ concentration produced by *P. aeruginosa* metabolism increased rapidly in the initial 7 days, while the pitting rate of the specimen during this period was as high as 0.170 mm/a, which was 1.85 times higher than that of the abiotic control. It is evident that NH_4_^+^ plays an important role in the acceleration of pitting of B30 copper–nickel alloy. Davalos-Monteiro (2019) immersed copper tubes in different concentrations of ammonia solution and found that the higher the concentration of ammonia, the faster the corrosion rate of copper tubes. NH_4_^+^ is easily hydrolyzed to produce NH_3_, which in turn reacts with available Cu+ ions to produce copper–ammonia complexes, which promote the dissolution of Cu (reaction 7), making the surface of the alloy brittle (Shalaby et al., 1999; Agarwal, 2002b). In addition, NH_3_ destroys the Cu_2_O film on the surface of the alloy, accelerating the pitting in B30 copper–nickel alloy (reaction 8).

## 5. Conclusion

It has been demonstrated by surface analysis and electrochemical testing that both extracellular electron transfer and the binding of the metabolite NH_3_ to copper are feasible mechanisms for the accelerated breakdown of passivation film and pitting of B30 copper–nickel alloy in seawater media containing *P. aeruginosa.*

## Data availability statement

The raw data supporting the conclusions of this article will be made available by the authors, without undue reservation.

## Author contributions

HL: conceptualization, investigation, data curation, and writing—original draft. MS and LM: methodology, data curation, and writing—review and editing. MD: funding acquisition, resources, supervision, and writing—review and editing. ZZ: experiments and data analysis. All authors contributed to the article and approved the submitted version.
